# Metagenome sequencing and 768 microbial genomes from cold seep in South China Sea

**DOI:** 10.1038/s41597-022-01586-x

**Published:** 2022-08-06

**Authors:** Huan Zhang, Minxiao Wang, Hao Wang, Hao Chen, Lei Cao, Zhaoshan Zhong, Chao Lian, Li Zhou, Chaolun Li

**Affiliations:** 1 Center of Deep Sea Research & CAS Key Laboratory of Marine Ecology and Environmental Sciences, Institute of Oceanology, Chinese Academy of Sciences, Qingdao, 266071 China; 2 Center for Ocean Mega-Science, Chinese Academy of Sciences, Qingdao, 266071 China; 3 University of Chinese Academy of Sciences, Beijing, 100049 China

**Keywords:** Marine biology, Environmental microbiology

## Abstract

Cold seep microbial communities are fascinating ecosystems on Earth which provide unique models for understanding the living strategies in deep-sea distinct environments. In this study, 23 metagenomes were generated from samples collected in the Site-F cold seep field in South China Sea, including the sea water closely above the invertebrate communities, the cold seep fluids, the fluids under the invertebrate communities and the sediment column around the seep vent. By binning tools, we retrieved a total of 768 metagenome assembled genome (MAGs) that were estimated to be >60% complete. Of the MAGs, 61 were estimated to be >90% complete, while an additional 105 were >80% complete. Phylogenomic analysis revealed 597 bacterial and 171 archaeal MAGs, of which nearly all were distantly related to known cultivated isolates. In the 768 MAGs, the abundant Bacteria in phylum level included Proteobacteria, Desulfobacterota, Bacteroidota, Patescibacteria and Chloroflexota, while the abundant Archaea included Asgardarchaeota, Thermoplasmatota, and Thermoproteota. These results provide a dataset available for further interrogation of deep-sea microbial ecology.

## Abstract (summary)

**Table:** 

Measurement(s)	metagenome assembled genomes
Technology Type(s)	metagenome sequencing and genome binning
Sample Characteristic - Organism	microorganism
Sample Characteristic - Environment	marine cold seep biome
Sample Characteristic - Location	South China Sea

## Background & Summary

Cold seeps are seafloor manifestations of methane-rich fluid migration from the sedimentary subsurface and support unique communities via chemosynthetic interactions fuelled^1^. The microorganisms inhabiting cold seeps transform the chemical energy in methane to products that sustain rich benthic communities around the gas leaks^2^. The use of next-generation sequencing methods has tremendously improved the insights into seep microbiomes and will advance microbial ecology from the diversity microbial distribution pattern to the adaptive survival strategy in deep-sea environments.

**Fig. 1:**
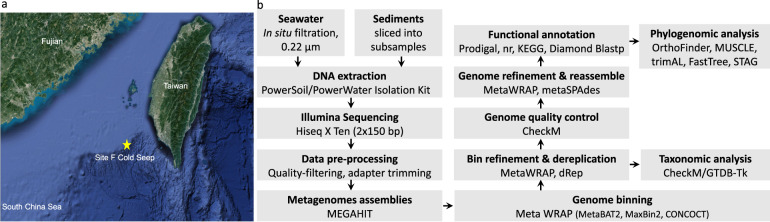
Sample collection and data analysis process. (**a**) Location and the sampling area in the cold seep field in the northern South China Sea. (**b**) Schematic overview of sampling and metagenomic analysis performed in this study. Each rectangle symbolizes processes containing descriptions (in bold), methods or tools used in the corresponding analysis.

**Fig. 2:**
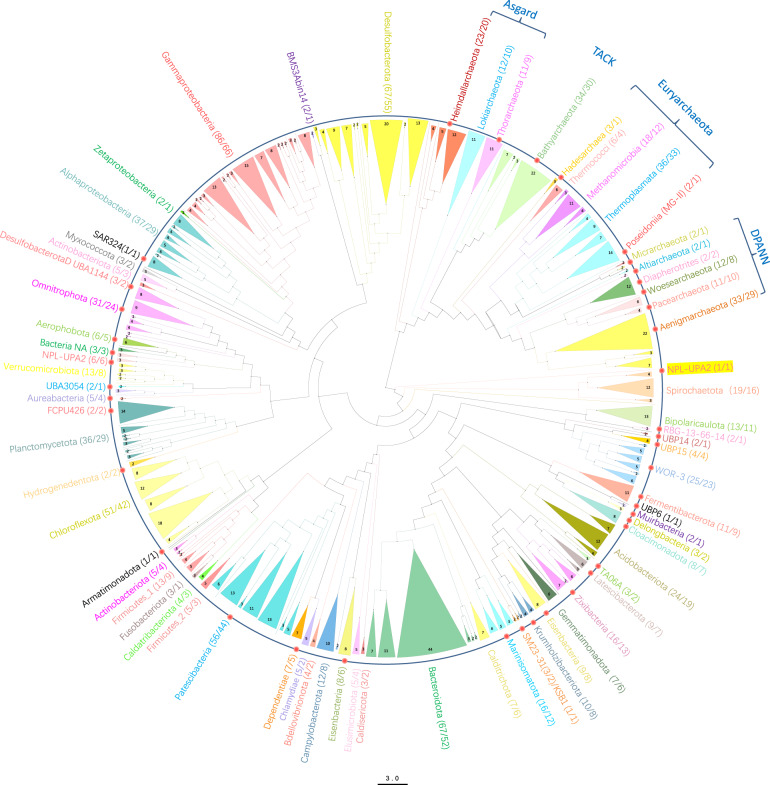
Phylogenetic diversity of 768 metagenome assembled genomes (MAGs) from cold seep in South China Sea (Supplementary Table 2) and reference genomes of Bacteria and Archaea available in RefSeq (Supplementary Table 3). The scale bar corresponds to 3.00 substitutions per amino acid position. The number of draft genomes in each node are provided. The branches with red dots have no cultured representatives.

The cold seep in Site F (also known as Formosa Ridge) is one of the active cold seeps on the north-eastern slope of the South China Sea (SCS)^3^, where the natural gas hydrate exposed on the seafloor and was covered by chemosynthetic communities mainly comprising deep-sea mussels and galatheid crabs^4^. The geochemical characters have been illustrated by the *in-situ* detection using the developed Raman insertion Probe (RiP) system and integrated sensors^5–7^. The horizontal and vertical variations in methane concentrations showed contrasting trends in fields from the center of flourishing communities to the margin of sediments^6^. No CH_4_ or H_2_S Raman peaks were detected in the cold seep fluids, while dissolved CH_4_ were identified in the fluids under the lush chemosynthetic communities, and the sediment pore water profiles collected near the cold seep were characterized by the loss of SO_4_^2−^ and increased CH_4_, H_2_S and HS^−^ peaks^5,7^. As the microbial communities in deep-sea cold seeps are often shaped by geochemical components in seepage solutions, we collected samples from the Site-F cold seep field in 2017, including the sea water closely above the invertebrate communities, the cold seep fluids, the fluids under the invertebrate communities and the sediment column around the seep vent (Fig. 1 and Table 1). The metagenomes were sequenced with Illumina HiSeq X Ten platform, with each metagenome yielding approximately 52.7 Gbps to 80.6 Gbps of clean bases (Table 2). We further obtained 768 metagenome-assembled genomes (MAGs) of environmental Bacteria and Archaea estimated to be >60% complete and <20% contamination (Supplementary Table 1). Of the MAGs, 61 were estimated to be >90% complete, while an additional 105 were >80% complete. There were 59 high-quality MAGs (completeness > 90% and contamination < 5%), accounting for 7.68% of the total. The anaerobic methanotrophic archaea (ANME), aerobic methanotrophic bacteria Methylococcales, sulfate-reducing Desulfobacterales, as well as sulfide-oxidizing Campylobacterales and Thiotrichales (Supplementary Table 2), well match the most favourable microbial metabolisms at methane seeps in terms of substrate supply. Meanwhile, the phylogenomic analysis suggests that this set of draft genomes includes highly sought-after genomes that lack cultured representatives, such as archaea Bathyarchaeota (30), Aenigmarchaeota (29), Heimdallarchaeota (20) and Pacearchaeota (10), and bacteria Patescibacteria (44), WOR-3 (23), Zixibacteria (13), Marinisomatota (12) and Eisenbacteria (6) *et al*. (Fig. 2). In addition, there are also some potential new phylum including NPL-UPA2 (7), UBP15 (4), FCPU426 (2) and SM23–31 (2) *et al*. All the non-redundant draft metagenome-assembled genomes described here were deposited into the National Center for Biotechnology Information (NCBI). These data will hopefully provide a resource for downstream analysis acting as references for largescale comparative genomics within globally vital phylogenetic groups, as well as allowing for the exploration of novel microbial metabolisms.

**Table 1:** Information for all samples utilized in this study.

Sample ID	Latitude	Longitude	Depth (m)	Sample	Environment characteristics	Sample collection date
SW_1	22.36	119.32	1119	seawater	water closely above the invertebrate communities	09/25/2017
SW_2	22.36	119.32	1121	seawater	water closely above the invertebrate communities	09/25/2017
SW_3	22.16	119.29	1127	seawater	cold seep fluids at gas plume	09/25/2017
SW_4	22.16	119.29	1120	seawater	fluid under the invertebrate communities	09/26/2017
RS_1	22.01	119.28	1153	sediment (0-2 cmbsf)	reductive sediments area	09/21/2017
RS_2	22.01	119.28	1153	sediment (2–4 cmbsf)	reductive sediments area	09/21/2017
RS_3	22.01	119.28	1153	sediment (4–6 cmbsf)	reductive sediments area	09/21/2017
RS_4	22.01	119.28	1153	sediment (6–8 cmbsf)	reductive sediments area	09/21/2017
RS_5	22.01	119.28	1153	sediment (8–10 cmbsf)	reductive sediments area	09/21/2017
RS_6	22.01	119.28	1153	sediment (10–12 cmbsf)	reductive sediments area	09/21/2017
RS_7	22.01	119.28	1153	sediment (12–14 cmbsf)	reductive sediments area	09/21/2017
RS_8	22.01	119.28	1153	sediment (14–16 cmbsf)	reductive sediments area	09/21/2017
RS_9	22.01	119.28	1153	sediment (16–18 cmbsf)	reductive sediments area	09/21/2017
RS_10	22.01	119.28	1153	sediment (18–20 cmbsf)	reductive sediments area	09/21/2017
RS_11	22.17	119.28	1121	sediment (0–20 cmbsf)	reductive sediments area	09/25/2017
RS_12	22.17	119.28	1121	sediment (20–55 cmbsf)	reductive sediments area	09/25/2017
RS_13	22.17	119.28	1121	sediment (55–90 cmbsf)	reductive sediments area	09/25/2017
RS_14	22.17	119.28	1121	sediment (90–125 cmbsf)	reductive sediments area	09/25/2017
RS_15	22.17	119.28	1121	sediment (125–160 cmbsf)	reductive sediments area	09/25/2017
RS_16	22.17	119.28	1121	sediment (160–195 cmbsf)	reductive sediments area	09/25/2017
RS_17	22.17	119.28	1121	sediment (195–230 cmbsf)	reductive sediments area	09/25/2017
RS_18	22.17	119.28	1121	sediment (230–265 cmbsf)	reductive sediments area	09/25/2017
RS_19	22.17	119.28	1121	sediment (265–300 cmbsf)	reductive sediments area	09/25/2017

**Table 2:** Metagenome sequencing statistics of each sample.

Sample ID	DNA Conc (ng/uL)	Volume (ul)	DNA Content (ug)	Total number of spots	Total number of bases	Q20 (Gbp)	Q20 (%)	Q30 (Gbp)	Q30 (%)	GC content (%)	Assembled contigs	N50 (bp)	Max contig length (kb)	Contigs ≥ 10-kb	BioProject accession No.	BioSample accession No.	SRA accession No.
SW_1	38.00	40.00	1.52	268,780,986	80,634,295,800	73.29	96.39	69.65	91.60	36.04	1,534,405	1,413	144.236	3,757	PRJNA707313	SAMN18200485	SRR13892607
SW_2	9.60	226.00	2.17	211,498,327	63,449,498,100	55.74	95.63	52.57	90.17	47.57	2,949,723	841	751.279	2,490	PRJNA707313	SAMN18200486	SRR13892606
SW_3	8.04	257.00	2.07	209,581,242	62,874,372,600	58.72	96.85	55.84	92.58	40.84	4,431,492	825	1533	4,192	PRJNA707313	SAMN18200487	SRR13892595
SW_4	14.96	254.00	3.80	213,078,219	63,923,465,700	59.35	96.40	56.37	91.57	42.30	4,784,748	875	755.276	11,638	PRJNA707313	SAMN18200488	SRR13892591
RS_1	6.30	80.00	0.50	210,376,373	63,112,911,900	59.24	96.49	56.22	91.57	45.43	5,827,198	718	452.39	5,238	PRJNA707313	SAMN18200489	SRR13892590
RS_2	9.90	92.00	0.91	210,044,424	63,013,327,200	59.52	96.63	56.58	91.87	45.19	5,772,932	698	427.971	3,591	PRJNA707313	SAMN18200490	SRR13892589
RS_3	5.94	135.00	0.80	197,222,088	59,166,626,400	57.00	96.09	53.80	90.69	47.52	6,485,322	686	274.684	4,051	PRJNA707313	SAMN18200491	SRR13892588
RS_4	3.54	135.00	0.48	175,717,792	52,715,337,600	55.41	96.60	52.62	91.73	47.43	6,321,787	684	341.334	4,586	PRJNA707313	SAMN18200492	SRR13892587
RS_5	5.18	87.00	0.45	210,273,664	63,082,099,200	59.68	97.12	57.00	92.76	45.76	6,627,379	693	387.906	6,193	PRJNA707313	SAMN18200493	SRR13892586
RS_6	6.66	84.00	0.56	210,496,319	63,148,895,700	59.64	96.67	56.73	91.95	44.75	6,083,188	645	336.042	4,020	PRJNA707313	SAMN18200494	SRR13892585
RS_7	5.48	90.00	0.49	210,215,804	63,064,741,200	59.48	96.59	56.50	91.75	45.59	6,621,563	714	542.774	7,236	PRJNA707313	SAMN18200495	SRR13892605
RS_8	6.44	88.00	0.57	221,216,994	66,365,098,200	62.28	96.48	59.09	91.53	44.89	6,417,883	706	236.738	7,174	PRJNA707313	SAMN18200496	SRR13892604
RS_9	4.59	90.00	0.41	217,513,891	66,365,098,200	61.87	97.17	59.13	92.87	45.50	6,677,947	720	408.486	8,524	PRJNA707313	SAMN18200497	SRR13892603
RS_10	4.01	139.00	0.56	205,597,241	61,679,172,300	58.32	97.12	55.71	92.76	46.23	6,469,960	695	434.319	6,316	PRJNA707313	SAMN18200498	SRR13892602
RS_11	4.48	140.00	0.63	225,785,488	67,735,646,400	65.71	98.12	63.58	94.95	43.50	5,201,517	758	415.221	6,919	PRJNA707313	SAMN18200499	SRR13892601
RS_12	5.44	71.00	0.39	232,763,725	69,829,117,500	67.64	98.08	65.40	94.85	43.42	3,094,173	796	635.758	5,801	PRJNA707313	SAMN18200500	SRR13892600
RS_13	4.88	131.00	0.64	225,203,094	67,560,928,200	65.44	98.14	63.39	95.07	42.51	3,500,917	817	383.492	5,980	PRJNA707313	SAMN18200501	SRR13892599
RS_14	5.16	135.00	0.70	241,881,543	72,564,462,900	70.34	98.21	68.19	95.21	42.45	1,869,638	683	305.012	1,746	PRJNA707313	SAMN18200502	SRR13892598
RS_15	5.70	135.00	0.77	225,763,481	67,729,044,300	65.60	98.17	63.53	95.07	42.59	1,863,140	671	305.23	1,759	PRJNA707313	SAMN18200503	SRR13892597
RS_16	5.66	135.00	0.76	236,000,139	70,800,041,700	68.61	98.12	66.40	94.97	42.71	1,738,136	837	239.392	4,010	PRJNA707313	SAMN18200504	SRR13892596
RS_17	5.12	135.00	0.69	259,045,539	77,713,661,700	74.83	97.86	72.04	94.20	42.40	2,671,409	805	268.822	4,707	PRJNA707313	SAMN18200505	SRR13892594
RS_18	9.10	71.00	0.65	245,684,586	73,705,375,800	71.51	98.29	69.37	95.35	42.72	2,997,732	793	322.724	4,926	PRJNA707313	SAMN18200506	SRR13892593
RS_19	7.88	68.00	0.54	219,631,776	65,889,532,800	63.85	98.29	61.94	95.34	42.01	2,343,571	779	213.639	3,431	PRJNA707313	SAMN18200507	SRR13892592

## Methods

### Sampling

**S**amples were retrieved from a cold seep field in the northern SCS by the KEXUE research vessel during the cruise in Sep 2017 (Fig. 1 a and Table 1). The water closely above the invertebrate communities was collected by an *in-situ* water sampling cylinder equipped on *FAXIAN* Remotely Operated Vehicle (ROV) during the dive 164 and 165 (sample ID: SW_1 and SW_2, respectively). The cold seep fluid was collected at the gas plumes during the dive 166 (sample ID: SW_3), and the fluid under the invertebrate communities was collected during the dive 167 (sample ID: SW_4). About 15 L water of each sample was filtered through a 0.22μm polycarbonate membrane (Millipore, Bedford, MA, USA). The membranes were stored at −80 °C and used for DNA extraction. A sediment core was collected by ROV at reductive sediments area nearby the invertebrate communities during dive 157. A thin outer layer ( < 1 cm) of the push core was discarded to avoid contamination. The black reduced sediment core, 20 cm in length, was sliced into layers by every two centimetres with a pushcore equipment (sample ID: RS_1 ~ RS_10). Another sediment core was collected at the same site by a deep-sea light weighted monitorable and controllable long-coring system^8^, and the sample layers of 0~300 cm below the seafloor (cmbsf) was collected from the sediment core and sliced into 35-cm subsamples (sample ID: RS_11 ~ RS_19). All subsamples were stored at −80 °C until DNA extraction. Environmental data (CH_4_, H_2_S and SO_4_^2−^) were detected *in situ* by a deep-sea laser Raman spectrometer mounted with the ROV in the previous report^5,9^.

### DNA extraction

A schematic overview of workflow in this study was shown in Fig. 1b. The genomic DNA from 2.5 g of each sediment subsamples was extracted using the PowerSoil DNA Isolation Kit (QIAGEN). The genomic DNA from the 0.22μm filters was extracted using the PowerWater DNA Isolation Kit (QIAGEN). The DNA were examined by gel electrophoresis, and the concentration of DNA was measured using Qubit® dsDNA Assay Kit in Qubit® 2.0 Flurometer (Life Technologies, CA, USA). OD value is between 1.8~2.0, DNA contents above 0.4 μg are used to construct library (Table 2).

### Metagenome sequencing

Metagenomic sequencing were performed at the Novogene (Tianjin, China) using the Illumina 2 × 150 PE protocols on an Illumina HiSeq X Ten platform. Preprocessing the Raw Data obtained from the sequencing platform using Readfq v8 (https://github.com/cjfields/readfq) was conducted to acquire the Clean Data for subsequent analysis. Clean Data of all 23 samples are available at NCBI Genbank (SRA) under the accession numbers SRR13892585~SRR13892607 (Table 2), and within the BioProject accession number PRJNA707313.

### Genome binning

The initial de novo assembly was carried out using MEGAHIT v1.1.3 with default parameters^10^. Short genomic assemblies ( < 1,000 bp) that could have biased the subsequent analysis were first excluded. Genomes were then binned based on their tetranucleotide frequency, differential coverage, and GC content, as well as codon usage, using different binning tools, including MetaBAT 2, MaxBin 2.0 and CONCOCT implemented by MetaWRAP v1.2.1 pipeline (default parameters) (Supplementary Table 1)^11–13^. The binning results were refined using the MetaWRAP package (parameters: -c 60 -x 20)^14^ and all the produced bin sets were aggregated and dereplicated at 95% average nucleotide identity (ANI) using dRep v2.3.2 (parameters: -comp 60 -con 20 -sa 0.9)^15^. Taxonomic classification of each bin was determined by CheckM v1.0.3 and GTDB-Tk with default parameters (Supplementary Table 2)^16,17^. The bin quality assessment (completeness > 60% and contamination < 20%) of different binners was then performed by CheckM v1.0.3 (parameters: lineage_wf)^17^. Next, the selected bins for each sample were reassembled by using metaSPAdes implemented through the MetaWRAP pipeline^14,18^. The coding regions of the final MAGs were predicted with the the Prodigal v2.6.3 (metagenome mode -p meta)^19^. All the predicted genes were searched against the nr database and KEGG prokaryote database using diamond blastp (parameters: -e 1e-5–id 40)^20,21^. Data of all MAGs are available at NCBI Assembly under the accession numbers JAGLBO000000000~ JAGMFB000000000 (Supplementary Table 1).

### Phylogenomic analysis

The 768 draft genomes and the 208 reference genome sequences accessed from NCBI GenBank (Supplementary Table 3) were combined to find orthologs for phylogenetic analysis by Orthofinder (default parameters)^22^. Each ortholog was aligned using MUSCLE v.3.8.31 (parameters:–maxiters 16)^23^, trimmed using trimAL v.1.2rev59 (parameters: -automated1)^24^ and manually assessed. Gene tree of each ortholog was constructed using FastTree v2.1.9 (parameters: -gamma -lg;)^25^. The final species tree was inferred based on 40,080 gene trees using STAG v1.0.0 (https://github.com/davidemms/STAG) and was viewed and annotated using FigTree v1.4.3 (http://tree.bio.ed.ac.uk/software/figtree/) (Fig. 2).

## Data Records

This project has been deposited at DDBJ/ENA/GenBank under the BioProject accession no. PRJNA707313, with the Sequence Read Archive deposited under the accessions SRR13892585~SRR13892607^26–48^. Other data is available through figshare^49^, including the fasta files containing the contigs of all 768 MAG, the newick format of the phylogenetic tree.

## Technical Validation

Potential contamination of samples was limited by following guidelines for analyses of microbiota communities^50,51^. Briefly, the samples were pre-treated in a sterile station in the lab of the Research Vessel *KEXUE*. DNA extractions took place within a dedicated laboratory space under a laminar flow hood using aseptic techniques (such as, surface sterilisation, DNA-OFF, use of sterile plasticware, and use of aerosol barrier pipette tips). Sample processing was completed within 2 days, using the same batch of PowerSoil DNA Isolation Kit for all sediment samples, and PowerWater DNA Isolation Kit for all water-filters samples. The filtered and trimmed Illumina reads were evaluated for their sequencing qualities using fastp v0.20.1 (https://github.com/OpenGene/fastp) with default parameters^52^. In all samples, the Q score for the reads of each sample was calculated and showed that more than 90% of reads scored Q30 (Table 2), indicating that most of the reads were constructed with low error rates. Metagenome data have been assembled and refined into MAGs using the automated quality control steps and assembly procedures described in the manuscript. To ensure the assembly quality of the contigs, several kmers (21,29,39,59,79,99,119,141) were selected in the assembly procedures of MEGAHIT. As for binning, more strict standards were selected, and the sequence after binning was re-assembled to ensure the best result.

## Supplementary information

Supplementary Table 1

Supplementary Table 2

Supplementary Table 3

## Data Availability

The above methods indicate the programs used for analysis within the relevant sections. The code used to analyse individual data packages is deposited at https://github.com/zhcosa/MAGs-from-cold-seep.
